# Characterization of post-vaccination SARS-CoV-2 T cell subtypes in patients with different hematologic malignancies and treatments

**DOI:** 10.3389/fimmu.2023.1087996

**Published:** 2023-04-28

**Authors:** Roald Pfannes, Arkadiusz Pierzchalski, Ambra Maddalon, Alexandra Simion, Christos C. Zouboulis, Gerhard Behre, Ana Claudia Zenclussen, Sabine Westphal, Stefan Fest, Gunda Herberth

**Affiliations:** ^1^Dessau Medical Center, Center for Oncology, Dessau, Germany; ^2^Department for Gastroenterology and Oncology, Diakonissenkrankenhaus Leipzig, Agaplession Mitteldeutschland GmbH, Leipzig, Germany; ^3^Department of Environmental Immunology, Helmholtz Centre for Environmental Research - UFZ, Leipzig, Germany; ^4^Department of Pharmacological and Biomolecular Sciences, University of Milan, Milan, Italy; ^5^Institute of Clinical Chemistry, Dessau City Hospital, Brandenburg Medical School Theodor Fontane, Dessau, Germany; ^6^Department of Dermatology, Staedtisches Klinikum Dessau, Brandenburg Medical School Theodor Fontane and Faculty of Health Sciences Brandenburg, Dessau, Germany; ^7^Department of Venereology, Staedtisches Klinikum Dessau, Brandenburg Medical School Theodor Fontane and Faculty of Health Sciences Brandenburg, Dessau, Germany; ^8^Department of Allergology, Staedtisches Klinikum Dessau, Brandenburg Medical School Theodor Fontane and Faculty of Health Sciences Brandenburg, Dessau, Germany; ^9^Department of Immunology, Staedtisches Klinikum Dessau, Brandenburg Medical School Theodor Fontane and Faculty of Health Sciences Brandenburg, Dessau, Germany; ^10^Department for Internal Medicine I, Dessau Medical Center and Brandenburg Medical School Theodor Fontane, Dessau, Germany; ^11^Perinatal Immunology Research Group, Medical Faculty, Saxonian Incubator for Clinical Translation (SIKT), University of Leipzig, Leipzig, Germany; ^12^Clinic of Pediatrics and Adolescent Medicine, Dessau City Hospital, Brandenburg Medical School Theodor Fontane, Dessau, Germany

**Keywords:** SARS-CoV-2 T cell subtypes, hematologic malignancies, myeloma, lymphoma, SARS-CoV-2 vaccine, CD4, Tfh cells, antigen-specific T cells

## Abstract

**Background:**

To evaluate the benefits of SARS-CoV-2 vaccination in cancer patients it is relevant to understand the adaptive immune response elicited after vaccination. Patients affected by hematologic malignancies are frequently immune-compromised and show a decreased seroconversion rate compared to other cancer patients or controls. Therefore, vaccine-induced cellular immune responses in these patients might have an important protective role and need a detailed evaluation.

**Methods:**

Certain T cell subtypes (CD4, CD8, Tfh, γδT), including cell functionality as indicated by cytokine secretion (IFN, TNF) and expression of activation markers (CD69, CD154) were assessed *via* multi-parameter flow cytometry in hematologic malignancy patients (N=12) and healthy controls (N=12) after a second SARS-CoV-2 vaccine dose. The PBMC of post-vaccination samples were stimulated with a spike-peptide pool (S-Peptides) of SARS-CoV-2, with CD3/CD28, with a pool of peptides from the cytomegalovirus, Epstein-Barr virus and influenza A virus (CEF-Peptides) or left unstimulated. Furthermore, the concentration of spike-specific antibodies has been analyzed in patients.

**Results:**

Our results indicate that hematologic malignancy patients developed a robust cellular immune response to SARS-CoV-2 vaccination comparable to that of healthy controls, and for certain T cell subtypes even higher. The most reactive T cells to SARS-CoV-2 spike peptides belonged to the CD4 and Tfh cell compartment, being median (IQR), 3.39 (1.41-5.92) and 2.12 (0.55-4.14) as a percentage of IFN- and TNF-producing Tfh cells in patients. In this regard, the immunomodulatory treatment of patients before the vaccination period seems important as it was strongly associated with a higher percentage of activated CD4 and Tfh cells. SARS-CoV-2- and CEF-specific T cell responses significantly correlated with each other. Compared to lymphoma patients, myeloma patients had an increased percentage of SARS-CoV-2-specific Tfh cells. T-SNE analysis revealed higher frequencies of γδT cells in patients compared to controls, especially in myeloma patients. In general, after vaccination, SARS-CoV-2-specific T cells were also detectable in patients without seroconversion.

**Conclusion:**

Hematologic malignancy patients are capable of developing a SARS-CoV-2-specific CD4 and Tfh cellular immune response after vaccination, and certain immunomodulatory therapies in the period before vaccination might increase the antigen-specific immune response. A proper response to recall antigens (e.g., CEF-Peptides) reflects immune cellular functionality and might be predictive for generating a newly induced antigen-specific immune response as is expected after SARS-CoV-2 vaccination.

## Introduction

Cancer patients undergoing immunosuppressive treatment have an increased risk of infection, including COVID-19. Additionally, due to cancer therapy and cancer-induced alterations at the immune system level, they might develop an impaired immune response to vaccination. In patients with hematologic malignancies, a sub-optimal humoral immune response to COVID-19 vaccines has been observed (1). The overall seroconversion rate in cancer patients after complete vaccination with one of the FDA-authorized COVID-19 vaccines was 94-98%, with a markedly lower rate in patients with hematologic malignancies (77-85%) versus solid tumors and healthy controls (98%) (2, 3). Thus, patients with hematologic malignancies need special attention during the COVID-19 pandemic. Due to the fact that the protection *via* specific antibodies after vaccination in these patients seems insufficient, a detailed analysis of specific cellular immune responses after vaccination is of the highest importance. A recent study addressed the cellular SARS-CoV-2 responses upon vaccination in healthy individuals, with a particular focus on the longevity and persistence of the SARS-CoV-2-specific T cell response versus the antibody immune response (4). TNF-α and IL-2 producing CD4+ T cells were identified as the hallmark of the vaccine-induced SARS-CoV-2-specific T cell response, and the cellular immune features were preserved across the analyzed time points. Furthermore, a stronger cellular immune response correlated with higher levels of spike-protein-specific IgG (4). Thus, while in healthy individuals, cellular immune responses correlate to specific antibody concentrations (5), the situation is apparently different in cancer patients. A recent study in which SARS-CoV-2-induced immune responses were analyzed after vaccination reports blunted cytokine production by peripheral blood cells after stimulation with SARS-CoV-2 peptides and reduced specific IgG blood concentration in immunocompromised patients compared to healthy controls, and this was dependent on the treatment (6). As shown in CD19-CART-treated B cell lymphoma patients, the ability to produce vaccine-induced antibody responses is lost. However, the same patients are still capable of developing an anti-SARS-CoV-2 T cell response (7). Thus, cellular immune responses are in of particular relevance for individuals and patients with a lower capacity to generate humoral responses after vaccination, as is the case in hematologic malignancies.

Therefore, in the present study, we analyzed SARS-CoV-2-specific T cell subtypes in detail after vaccination of patients with hematologic malignancies and age-matched healthy controls. Multi-parameter flow cytometry data were complemented by antibody titers. Particularly, the reactivity of CD4, CD8, Tfh and γδT cells to SARS-CoV-2 spike peptides (S-Peptides) was analyzed in post-vaccination samples *in vitro*. CD3/CD28 stimulation was used as a control for general T cell reactivity, and a pool of peptides from the cytomegalovirus, Epstein-Barr virus and influenza A virus (CEF-Peptides) was used as a control for specific reactivity to peptides other than SARS-CoV-2. Finally, specific immune responses after vaccination were compared between patients and controls and patients’ immune responses were analyzed in regard to clinical data.

## Methods

### Study participants

In this observational study, patients (N=12) with a known hematologic malignancy and with two doses of SARS-CoV-2 vaccines were recruited in the Dessau Medical Center, Brandenburg Medical School Theodor Fontane, Dessau, Germany, during the regular visits for their control or cancer therapy from January to June 2021. Patients diagnosed with multiple myeloma (MM), follicular lymphoma (FCL), chronic lymphocytic leukemia (CLL) or mantle cell lymphoma (MCL) were included regardless of their clinical status (stable, progress, remission). Patients had no or different therapies and active therapy was defined as active cancer therapy during the month before the start of vaccination. Sex- and age-matched healthy controls (N=12) with two doses of SARS-CoV-2 vaccines were randomly recruited among the hospital’s healthcare workers, laboratory staff and volunteers. Blood was collected from all patients and controls after the second SARS-CoV-2 vaccine dose and used for the analysis of SARS-CoV-2-specific T cell subtypes and in patients also for specific antibody concentrations. All patients and healthy controls gave informed consent for participation in this study. The study was approved by the Ethics Committee of the Medical Association of Saxony Anhalt (#16/21).

### Blood collection and PBMC isolation

Heparinized whole blood was collected from patients and controls by venipuncture in a range of 5–10 ml. Peripheral blood mononuclear cells (PBMC) were isolated by gradient centrifugation on Ficoll-Paque PLUS (GE Healthcare; Chicago, IL, USA). PBMCs were gradually frozen in fetal bovine serum (FBS) with 10% dimethyl sulfoxide (DMSO) at −80°C using a cell freezing device. Cells were stored at −150°C until use for the identification of specific T cell subtypes.

### Immune cell stimulation assay

For the identification of SARS-CoV-2-specific T cells, we used three peptide pools of 15-mer peptides with 11-amino acid overlap covering the whole spike proteins: PepTivator SARS-CoV-2 Prot_S, _S1 and _S+ (all Miltenyi Biotec, Bergisch Gladbach, Germany; for convenience we refer to the three pooled peptide pools, S-Peptides). The peptides used as stimuli in this assay can be presented on MHC class I and II to activate spike-peptide-specific CD8+ T cells and CD4+ T cells, respectively. For the analysis of T cell responses against microbial antigens other than SARS-CoV-2, a pool of 32 peptides, 8-12 amino acids in length, with sequences derived from the human cytomegalovirus (CMV), Epstein-Barr virus (EBV) and influenza A virus was used (AnaSpec, Fremont, CA, USA; referred as CEF-Peptides). PBMCs were thawed at 37°C, and residual DMSO was removed by washing in IMDM with 10% FBS. After trypan blue staining, 1 x 10^6^/100 µL of live PBMC (IMDM Sigma-Aldrich, St. Louis, MI, USA) containing 2% human serum were seeded into 96-well plates and allowed to rest overnight at 37°C and 5% CO2. After this time, PBMCs were stimulated with 1 µg/ml of the pooled S peptides, CEF peptides (1µg/ml) or CD3/CD28 beads (bead:cell ratio 1:4) or left unstimulated in IMDM (2% human serum) in a total volume of 200 µL for 6 h. Brefeldin A (Sigma-Aldrich, St. Louis, MI, USA) was added 2 h later to capture cytokines and activation markers inside the cells for intracellular staining. PMBCs were harvested and stained for multi-parameter flow cytometry.

### Flow cytometric analysis

**Figure 1:**
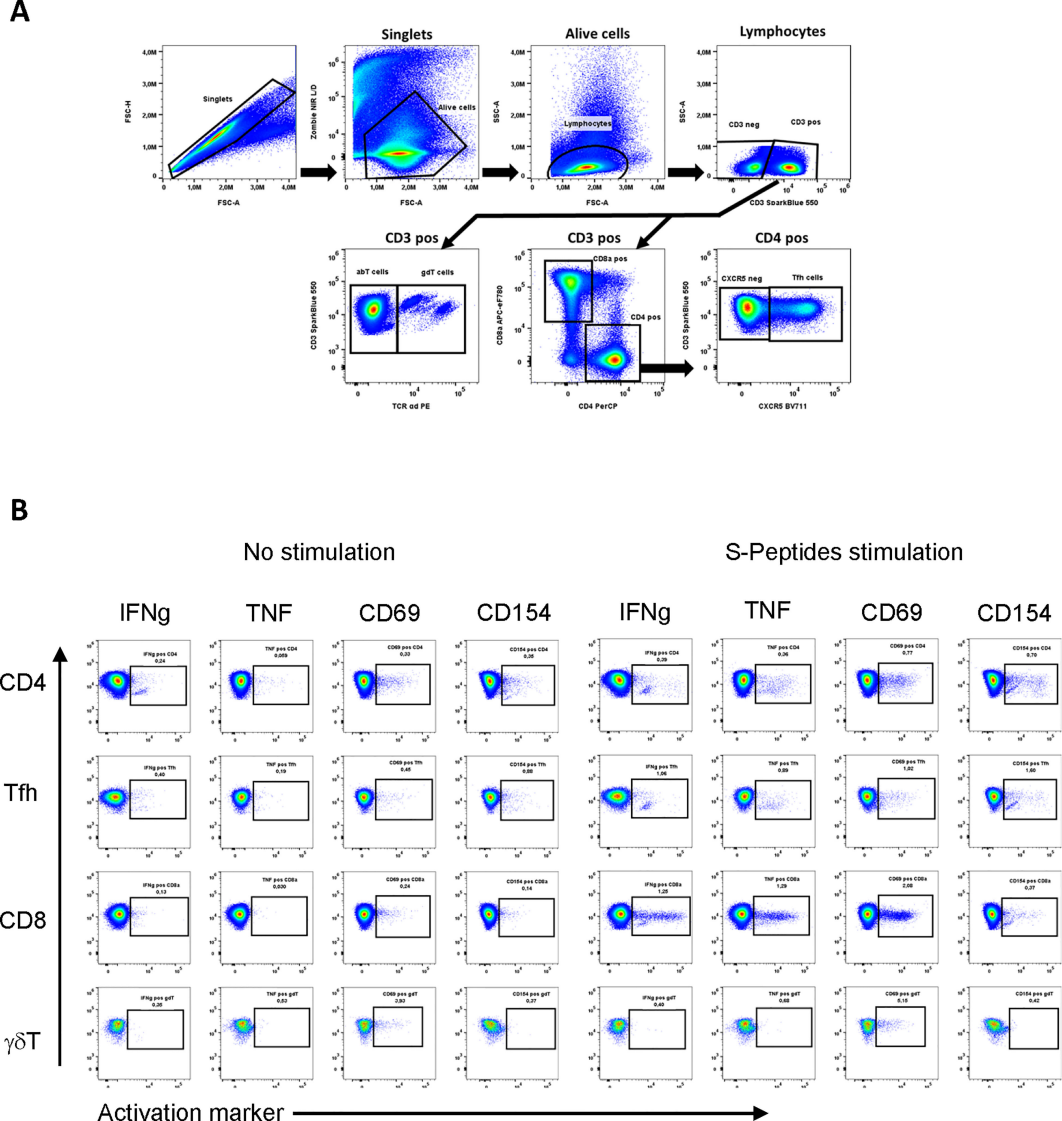
Flow cytometric analysis of SARS-CoV-2-specific T cells after two vaccine doses. Gating strategy **(A)** and exemplary dot plots **(B)** of unstimulated and stimulated samples from one control subject. Cells of interest (CD4, CD8, Tfh, γδT) were gated as follows: After doublet and dead cell exclusion, lymphocytes were gated based on FSC-A/SSC-A properties. CD4+ (Th cells) and CD8a+ (Tc cells) T cells were identified out of the CD3+ gate. CXCR5+ (Tfh cells) were identified in CD3+CD4+ cells. In CD3+ cells, cells expressing TCRγδ were defined as γδTCR+ (γδT cells). The same gating strategy has been applied for t-SNE analysis. T cells specific for the spike protein of SARS-CoV-2 were identified after 6 h stimulation of total PBMC using overlapping peptides (S-Peptides), covering the complete sequence of the spike protein. After intracellular staining of cytokines and activation markers, activated T cell subtypes were identified by the production of IFN and TNF or the expression of the activation markers CD69 or CD154 *via* flow cytometry **(B)**.

After washing in phosphate-buffered saline without Ca^2+^ and Mg^2+^ (PBS), dead cells in PBMC were discriminated by Zombie NIR Dye (BioLegend, San Diego, CA, USA) staining. PBMCs were stained with antibodies (**Supplementary Table S1**) in two steps. In the first step, only 2 surface antibodies of cocktail A were applied in 1% FBS in PBS for 10 min at room temperature (RT). Then, additional 5 surface antibodies (cocktail B) were used in 1% FBS in PBS for 20 min at RT. Cells were fixed in a BD Lysing solution (BD Biosciences, Haryana, India) for 10 min at RT and permeabilized using a BD Perm2 solution (BD Biosciences) for 10 min at RT. The remaining markers were stained intracellularly in 1% FBS in PBS (antibody cocktail C) for 20 min at RT. Cytometric analysis was performed on CYTEK Aurora (CYTEK Biosciences, Fremont, CA, USA). Data analysis was performed with FCS Express 7 software (*De Novo* Software, Pasadena, CA, USA). The gating strategy is presented in **Figure 1**.

### t-SNE analysis of T cell subtypes

Following flow cytometrical analysis of S-Peptides-stimulated PBMC, a further analysis using the t-distributed stochastic neighbor embedding (t-SNE) transformation tool present on FCS Express 7 was performed. The FCS files of controls and patients were merged and, after manual gating of T cell subtypes CD3, CD4, CD8, Tfh and γδT (**Figure 1A**), the results were visualized in 2D t-SNE maps. Briefly, a sample size of 20,000 total events was selected to allow the full representation of each T cell subpopulation. T-SNE was run with the down-sampling algorithm as an interval, with an iteration number of 500, perplexity of 50 and approximation of 0.5. Furthermore, the optimized t-SNE was selected, and the plot was also estimated for unsampled events. After obtaining the 2D map, group gating (controls, patients, patients with/without treatment, disease category) and single gating were performed by sample ID.

### SARS-CoV-2 serology

For SARS-CoV-2 serology, patient samples were collected in serum gel tubes. Serum samples derived from these tubes were stored temporarily at 4°C for testing, performed usually within 2 days of collection, and were frozen at −80°C for longer-term storage. All samples were obtained *via* a quality assurance protocol, which qualified for an institutional review board waiver, and no patient identifiers were used. The antibody concentrations were measured with the Roche Elecsys S-Ab assay and its predecessor, the Roche Elecsys anti-nucleocapsid antibody (N-Ab) assay (Roche Diagnostics, Mannheim, Germany). Both are 1-step, double-antigen sandwich electrochemiluminescent immunoassays that detect total amounts of IgG, IgM, and IgA antibodies against SARS-CoV-2 spike (S) and nucleocapsid (N) antigens, respectively. The S-Ab test is a semiquantitative assay with an analytical measurement range (AMR) claim of 0.4 to 250 U/mL; positive results are defined as concentrations at or greater than 0.8 U/mL. In contrast, the N-Ab assay is a qualitative assay that uses a calibrator-based cutoff index (COI) at or greater than 1.0 as the definition of positivity/reactivity. Both S-Ab and N-Ab assays were performed in the cobas e602 module of the Roche cobas 6000 total automation system (Roche Diagnostics, Mannheim, Germany). Serum samples of controls were not available.

### Statistical analysis

All data was analyzed for normal distribution before statistical analysis. Regarding T cell subpopulations, calculations in this study are based on the percentage of cells (CD4, CD8, Tfh, γδT) being positively gated for the production of interferon (IFN), tumor necrosis factor (TNF) and the expression of CD69 and CD154, all used as an activation marker. Data showing these activated T cell subpopulations is presented as pie charts (as a percentage of activated T cell subtypes when all activated T cells are regarded as a whole in the certain stimulation type) or as box plots showing the median, interquartile range (IQR) and minimum-to-maximum whiskers of the percentage of activated cells of the parent cell population. The comparison between groups was performed with the non-parametric Mann-Whitney U Test. The association between certain T cell subtypes was analyzed by using the non-parametric Spearman’s rank correlation test. The correlation coefficients from these tests are presented in heat maps. A probability value of P < 0.05 was considered significant. All analyses have been carried out using Statistica Version 13.3 (StatSoft, Hamburg, Germany) or GraphPad Prism 9.

## Results

### Characteristics of the study population

The characteristics of the study population are shown in **Table 1**. Included patients were suffering from multiple myeloma (n=6), follicular lymphoma (n=3), chronic lymphocytic leukemia (n=2) and mantle cell lymphoma (n=1). The disease activity at vaccination was stable in one patient, progressive in five patients and remissive in six patients. All study participants received two doses of the same SARS-CoV-2 vaccine; patients BNT162b2 (n=11) and AZD1222 (n=1) and controls BNT162b2 (n=12). Four patients had an active anticancer therapy with ibrutinib, lenalidomide, daratumumab-lenalidomide-dexamethasone, and bortezomib, respectively, during the month before the start of vaccination. Therapy of patients treated before this period and starting after the second vaccine dose (in one patient after the first) included rituximab + chemotherapy, rituximab + venetoclax, chemotherapy + bortezomib, ibrutinib, daratumumab + chemotherapy + bortezomib. Two patients were under observation without treatment. Healthy controls (N=12) were matched by age and sex with the patient’s group and received two doses of the BNT162b2 vaccine (**Table 1**). Neither patients nor controls experienced a SARS-CoV-2 infection prior to vaccination and until T cell analysis. In patients, anti-nucleocapsid antibody measurements were performed and no reactivity was found (data not shown).

**Table 1:** Study participant characteristics.

Patient Characteristics	N (%)
Total	12 (100%)
Age, years, (median, IQR)	61 (55-70)
Sex	
Male	7 (58%)
Female	5 (42%)
Type of cancer	
Multiple Myeloma (MM)	6 (50%)
Follicular Lymphoma (FCL)	3 (25%)
Chronic Lymphocytic Leukemia (CLL)	2 (16.7%)
Mantle Cell Lymphoma (MCL)	1 (8.3%)
Disease status at vaccination	
Stable disease	1 (8.3%)
Remission (clinical and VGPR*)	6 (50%)
Progress	5 (42%)
Active therapy#	
yes	4 (33.3%)
no	8 (66.7%)
Type of active therapy#	
Ibrutinib (mono)	1 (8.3%)
Lenalidomid	1 (8.3%)
Daratumumab + Lenalidomide + Dexamethasone	1 (8.3%)
Bortezomib	1 (8.3%)
Type of last anti-cancer therapy§	
Rituximab + chemotherapy	1 (8.3%)
Rituximab + Venetoclax	1 (8.3%)
Chemotherapy + Bortezomib	2 (16.7%)
Ibrutinib (mono)	1 (16.7%)
Daratumumab + chemotherapy + Bortezomib	1 (8.3%)
Never treated/no active therapy	2 (16.7%)
SARS-CoV-2 vaccine	
BNT162b2	11 (91.7%)
AZD1222	1 (8.3%)
Days between second vaccine and T cell analysis (median, IQR)	49 (27.5-90)
Control Characteristics	
Total	12 (100%)
Age, years, (median, IQR)	60.5 (55-67.5)
Sex	
Male	5 (42%)
Female	7 (58%)
Vaccine type BNT162b2	12 (100%)
Days between second vaccine and T cell analysis (median, IQR)	81 (74-105)

*VGPR=very good partial remission according to IMWG-criteria.

Active therapy means therapy before and during the vaccination period. Therapy started a minimum one month before 1st vaccination and ended more than one month after 2nd vaccination.

§ Last therapy includes patient therapies more than one month before 1st dose and starting after the 2nd vaccine dose and, in one patient, after the 1st dose.

### Identification of SARS-CoV-2-specific and other T cell subtypes

**Figure 2:**
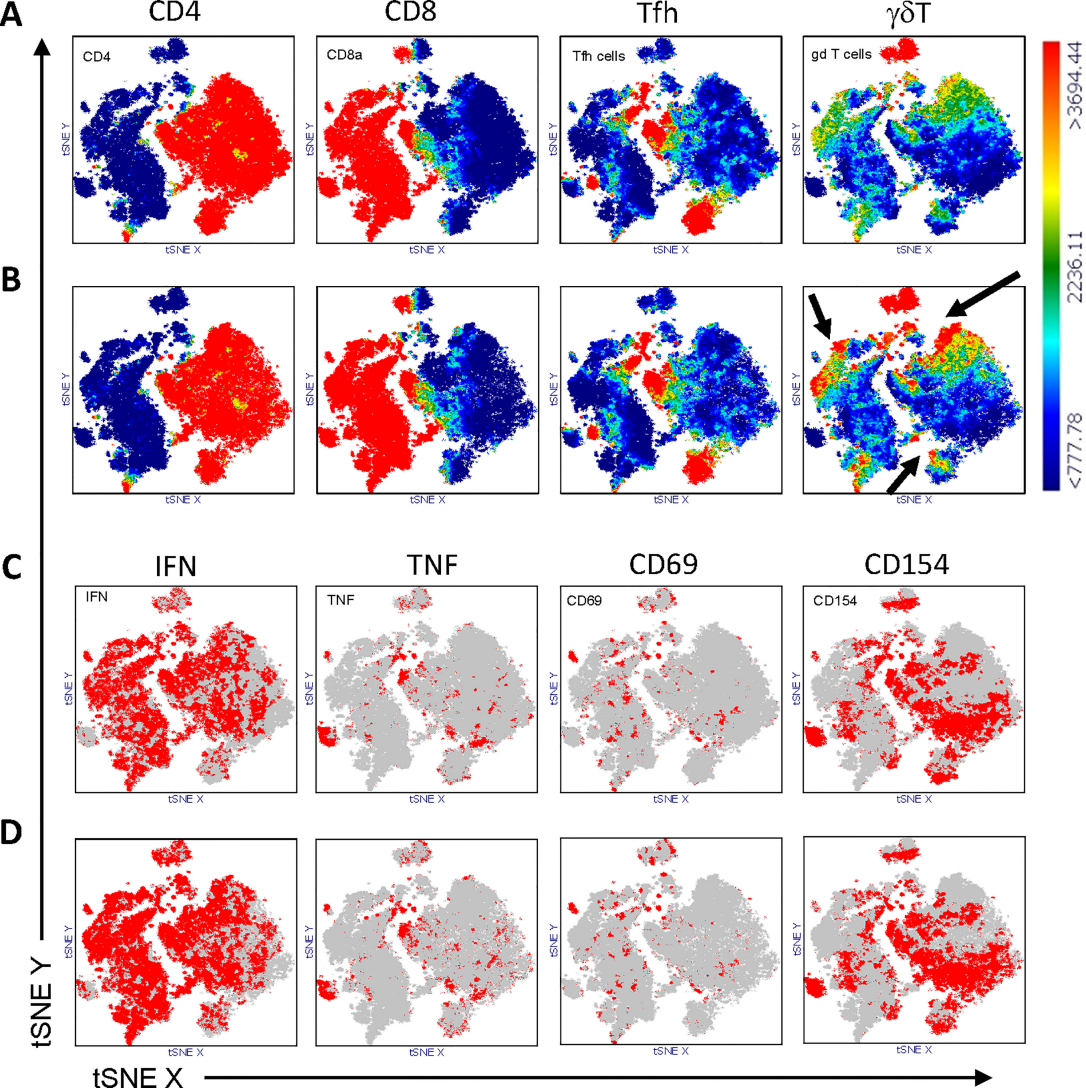
Distribution of T cell subtypes within CD3+ T cells in controls **(A)** and patients **(B)** and distribution of S-Peptides-activated cells in controls **(C)** and patients **(D)**. T cells from controls (N=12) and patients (N=12) were concatenated and subjected to unsupervised analysis by t-distributed stochastic neighbor embedding (t-SNE), color-coded for the expression of CD4, CD8, CXCR5 (Tfh cells), γδTCR (γδT cells) from blue (low) to red (high), **(A, B)** and for the expression of IFN, TNF, CD69 and CD154 from grey (<1000) to red (>1000), **(C, D)**. To be noted, the higher abundance of γδT cells in patients **(B)** compared to controls **(A)** is highlighted by arrows.

Multi-parameter flow cytometry was performed to identify T cell subtypes. Cell populations were gated, as shown in **Figure 1A**. To analyze SARS-CoV-2-specific T cell responses after vaccination, we stimulated PBMCs isolated from patients and controls with a SARS-CoV-2 spike-peptide pool (S-Peptides). Cells left unstimulated or stimulated with CD3/CD28 beads were used as controls. For stimulation efficacy for viruses other than SARS-CoV-2, cells were stimulated with a pool of CEF peptides (CEF-Peptides). For the present study, four distinct T cell subtypes were considered since these cells are able to react to peptide stimulation: CD4, CD8, T follicular helper cells (Tfh) and gamma-delta T cells (γδT). The ability to produce IFN-gamma (IFN) and TNF-alpha (TNF) as well as the expression of the activation markers CD69 and CD154 after stimulation were used to identify the frequency of specifically activated cells, which are used for the calculations in this study. A representative dot plot of the staining showing a control sample without stimulation and after S-Peptides stimulation is depicted in **Figure 1B**. Representative dot plots for the stimulation with CEF-Peptides and CD3/CD28 from the same control are shown in **Supplementary Figure S1**. Furthermore, in S-Peptides-stimulated samples from patients and controls an unsupervised analysis of T cell subtype distribution and activation was performed by using t-distributed stochastic neighbor embedding (t-SNE), **Figure 2**. It was visible that in patients (**Figure 2A**), compared to controls (**Figure 2B**), γδT cells as measured by TCRγδ expression were distributed within the compartments of CD4, CD8 and Tfh cells (indicated by arrows). The analysis of activation by S-Peptides in the T cell subtypes revealed higher activation levels in patients, at least for IFN and CD154 (**Figure 2D**) compared to controls (**Figure 2C**).

### Hematologic cancer patients have higher frequencies of SARS-CoV-2-specific T cells after vaccination compared to healthy controls

**Figure 3:**
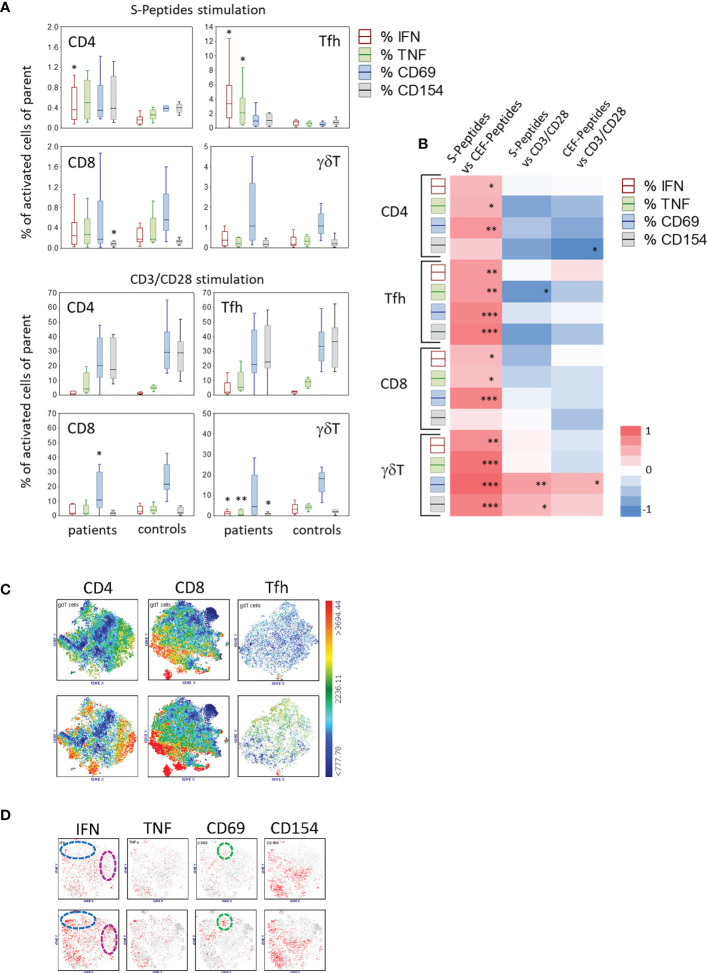
Activated T cell subtypes **(A)** in S-Peptides and CD3/CD28 stimulated samples of patients (N=12#) and controls (N=12) after two vaccine doses. Box plots with median, interquartile range and minimum-to-maximum whiskers show activated T cell subtypes as a percentage of the parent population (CD4, Tfh, CD8, γδT). Cells were identified after 6 h stimulation of PBMC and intracellular staining of cytokines (IFN, TNF) and activation markers (CD69, CD154) followed by flow cytometry. Data analysis with Mann-Whitney U Test, *p<0.05, **p<0.005. Please note the different scaling for better visibility. # Due to the low blood amount, the CD3/CD28 stimulation analysis was not performed in one patient. **(B)** Correlation between the particularly activated T cell subtypes in patients and controls. Activated T cell subtypes represent the percentage of parent cells producing IFN, TNF, CD69 or CD154 after stimulation with S or CEF-Peptides or CD3/CD28. A heat map presents the correlation coefficients (R) gained from the Spearman Rank correlation test, red and blue colors indicate positive and negative correlations, respectively, *p<0.05, **p<0.005, ***p<0.0005. **(C)** Distribution of γδT cells in the population of CD4, CD8 and Tfh cells as shown by t-SNE analysis color-coded for the expression of TCRγδ from blue (low) to red (high) in controls (top) and patients (bottom). **(D)** t-SNE analysis of activation markers in γδT cells in controls (top) and patients (bottom). Color-coded from grey (<1000) to red (>1000) with dotted circles indicating the area of prominent t-SNE plot shift.

The frequencies of reactive T cell subtypes, as a percentage of the parent cell population, differed between patients and controls, independently of the stimulus. The highest values for S-Peptides stimulation were measured in patients, median (IQR), 3.39 (1.41-5.92) and 2.12 (0.55-4.14) for IFN- and TNF-producing Tfh cells compared to controls, 0.85 (0.39-0.99) and 0.63 (0.35-0.85), respectively (**Figure 3A**; **Supplementary Tables S2, S3**). Also, the frequency of IFN-producing CD4+ T cells was higher in patients, 0.36 (0.17-0.80), compared to controls, 0.16 (0.09-0.22). Thus, some patients unexpectedly developed a stronger anti-SARS-CoV-2 cellular immune response after vaccination compared to controls (**Figure 3A**; **Supplementary Tables S2, S3**). However, the frequency of CD8+CD154+ cells was slightly lower in these patients compared to controls (**Figure 3A**). Of note, in patients the distribution of the analysed T cell subtypes was not related to their sex (**Table S8**). In controls, male subjects had a lower percentage of SARS-CoV-2-specific CD154+ γδT cells and a higher percentage of unstimulated IFN-producing γδT cells (**Table S7**). Thus, the comparison of these T cell subtypes between patients and controls might be biased by sex distribution and needs to be interpreted with caution. Other potential impacting factors, i.e. disease state, age, time interval between second vaccine dose and T cell analysis were no, or only marginally associated to T cell responses (see **Supplementary Material** and **Supplementary Tables S6–S12**).

As expected, CD3/CD28 stimulation, used as a positive control for T cell reactivity, led to the highest percentage of activated T cell subtypes. The frequency ranges for CD4+ T cells expressing CD69 or CD154 were similar in patients and controls, being 20.33 (median; IQR (12.12-39.22) and 17.47 (11.34-39.34) in patients, respectively (**Supplementary Tables S2, S3**). However, the response to CD3/CD28 stimulation in patients was lower for CD8+CD69+ T cells and for IFN-, TNF- and CD154-producing γδT cells (**Figure 3A**). In unstimulated samples, the frequency of baseline-activated T cells was very low (**Supplementary Tables S2, S3**). Nevertheless, a significant difference was seen in CD4+ IFN-producing and in IFN- and TNF-producing Tfh cells, these activated T cell subtypes being at a higher frequency in patients (**Supplementary Figure S2A**). Also, the frequency of T cells responding to CEF-Peptides stimulation was higher in patients, compared to controls, at least for IFN- and TNF-producing CD4+ and Tfh cells (**Supplementary Figure S2A**).

In both vaccinated patients and controls, the percentages of antigen-specific (S- and CEF-Peptides) activated T cell subtypes correlated positively with each other. This leads to the assumption that vaccinated individuals who developed a strong T cell response to SARS-CoV-2 peptides had pre-existing immune responses to antigens covered in the CEF-Peptides pool due to previous infections with these germs in their life (**Figure 3B** and **Supplementary Figure S2B**, shown for patients and controls separately). In contrast, a negative association was found between peptide-stimulated cells and the percentage of cells responding to CD3/CD28 stimulation (**Figure 3B**). One exception here was in the correlation between CEF-Peptides-stimulated γδT cells vs. CD3/CD28-stimulated cells, which was positive in patients compared to controls (**Supplementary Figure S2B**).

Of note was the difference in the distribution of γδT cells between patients and controls in S-Peptides-stimulated samples seen by t-SNE analysis (**Figures 2A, B**). We therefore analyzed the phenotype of these cells in more detail. In controls, a subpopulation of CD8 T cells expressing TCRγδ was detected; whereas in patients, subpopulations of CD4, CD8 and Tfh cells expressing TCRγδ were found (**Figure 3C**, bottom). Regarding the activation of γδT cells (**Figure 3D**), a higher abundance was observed in patients’ samples, at least for IFN (at the bottom in the cluster blue circle 23%, cluster pink circle 10%, versus the corresponding clusters in controls 14.7% and 3.7% at top). Similar for CD69, as seen in the unbiased comparative t-SNE plot analysis, the abundance difference between patients and controls in the cluster green circle was 9.7% vs. 4% (**Figure 3D**).

### Active therapy before vaccination was associated with a higher SARS-CoV-2-specific T cell response

**Figure 4:**
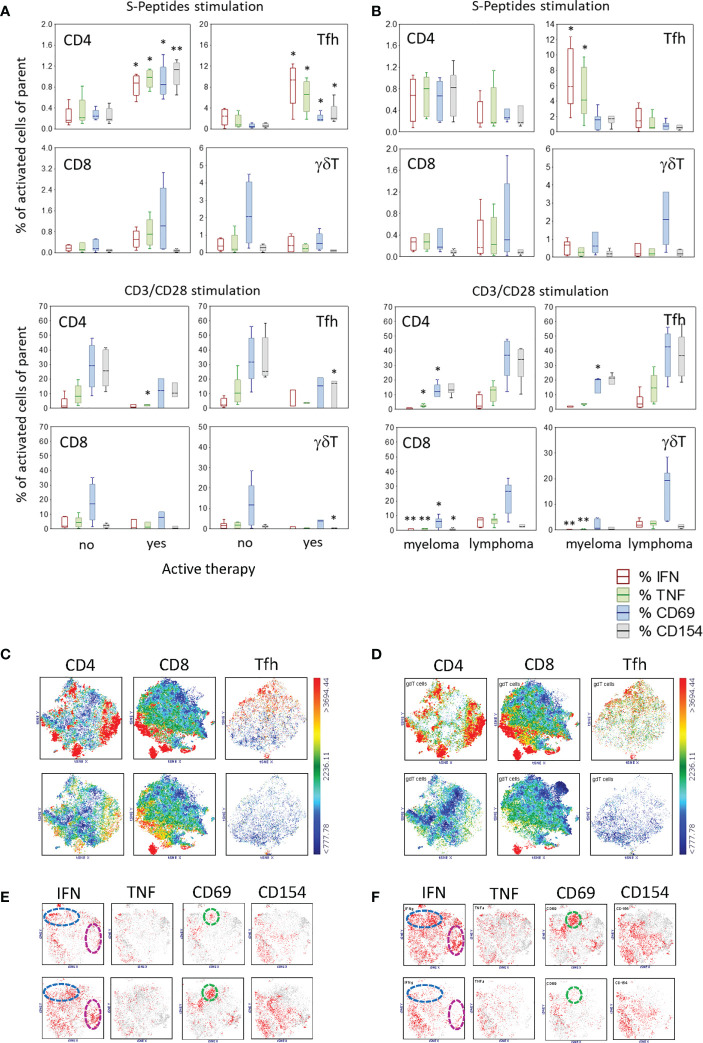
Activated T cell subtypes after S-Peptides or CD3/CD28 stimulation in samples of patients regarding **(A)** active therapy (no, N=8#, yes N=4) and **(B)** disease category (myeloma, N=6#, lymphoma, N=6) after two vaccine doses. Box plots with median, interquartile range and minimum-to-maximum whiskers show activated T cell subtypes as a percentage of the parent population (CD4, Tfh, CD8, γδT). Cells were identified after 6 h stimulation of PBMC and intracellular staining of cytokines (IFN, TNF) and activation markers (CD69, CD154) followed by flow cytometry. Active therapy means therapy before and during the vaccination period. Therapy started a minimum one month before 1st vaccination and ended more than one month after 2nd vaccination. Data analysis with Mann-Whitney U Test, *p<0.05, **p<0.005. Please note the different scaling for better visibility. # Due to the low blood amount, the CD3/CD28 stimulation analysis was not performed in one patient. **(C)** Distribution of γδT cells in the population of CD4, CD8 and Tfh cells as shown by tSNE analysis color-coded for the expression of γδTCR from blue (low) to red (high) in patients with no (top) and with active therapy (bottom) and similarly in **(D)** myeloma patients (top) and lymphoma patients (bottom) **(E)** t-SNE analysis of activation markers in γδT cells in patients with no (top) and with active therapy (bottom). **(F)** t-SNE analysis of activation markers in γδT cells in patients with myeloma (top) and with lymphoma (bottom). Color-coded from grey (<1000) to red (>1000) with dotted circles indicating the area of prominent t-SNE plot shift.

To determine whether therapy before and during vaccination had an impact on the development of a specific immune response to SARS-CoV-2 vaccination, patients were categorized into two groups: with therapy during the month before first vaccination and without therapy in this period. Of the 12 participating patients, four had an active therapy (i.e., ibrutinib, lenalidomide, daratumumab-lenalidomide-dexamethasone, bortezomib), and eight were without therapy. The frequency of SARS-CoV-2-specific T cells was highly increased in all CD4+ and Tfh cell compartments in patients with therapy (**Figure 4A**). However, the general reactivity of T cells measured by CD3/CD28 stimulation was, by means of frequency, less in these patients, being significantly reduced for CD4+TNF+, Tfh+CD154+ and γδT+CD69+ cells (**Figure 4A**). In contrast, there was no difference in unstimulated cells in these two patient groups (**Supplementary Figure S3A**). Regarding the reactivity to CEF-Peptides, patients with an active therapy had higher frequencies of CD8+ T cells producing IFN, TNF or CD69 after stimulation with these peptides (**Supplementary Figure S3A**).

Because γδT cells differed in their abundance, being higher in patients compared to controls, we analyzed the distribution and activation of these cells regarding the therapy in the two patient groups. The unbiased t-SNE analysis revealed that the expression of TCRγδ was co-localized with CD4, CD8 and CXCR5 in patients having no therapy prior to vaccination (**Figure 4C**, top), whereas in patients with active therapy, the abundance of these cells was reduced (**Figure 4C**, bottom). However, in patients with active therapy prior to vaccination, activated γδT cells seem to be more abundant, as seen by the expression of IFN (no 21% vs. yes 26%, blue circle cluster; no 5% vs. yes 14.2%, pink circle cluster) and CD69 (no 4.3% vs. yes 14.2%, green circle cluster) (**Figure 4E**).

### Compared to lymphoma patients, myeloma patients had a higher frequency of SARS-CoV-2-specific Tfh cells after vaccination and a shift in γδT cells

In order to understand whether the type of hematologic malignancy is a relevant factor for the establishment of a specific SARS-CoV-2 immune response after vaccination, patients were grouped into two categories: myeloma (i.e., multiple myeloma, n=6) and lymphoma (including follicular lymphoma, chronic lymphocytic leukemia and mantle cell lymphoma, n=6). Stimulation with SARS-CoV-2-specific peptides revealed that myeloma patients had an increased percentage of Tfh cells reacting to these peptides with the production of IFN and TNF compared to lymphoma patients (**Figure 4B**). In general, myeloma patients showed a reduced T cell activation *via* CD3/CD28 compared to lymphoma patients, especially for IFN- and TNF-producing T cells (**Figure 4B**). Excepting a higher frequency of CD4+IFN+ T cells in unstimulated samples in myeloma patients, there were no other differences regarding unstimulated and CEF-Peptides- stimulated samples in the two patient groups (**Figure S3B**). Regarding the disease state of patients, data is presented in **Supplementary Results** and **Table S10**.

When we observed that patients had a different distribution of γδT cells compared to controls (**Figures 3A, B**), our next question was whether this distribution is related to the disease category. With the unbiased t-SNE plot analysis, it was visible that γδT cells were more abundant in myeloma patients, TCRγδ being co-expressed in part with CD4, CD8 or in the compartment of Tfh cells by co-expressing CXCR5 (**Figure 4D**). Compared to lymphoma patients, the abundance of activated γδT cells was higher in myeloma patients (**Figure 4F**).

### SARS-CoV-2-specific T cells do not correlate with antibody concentrations in patients

**Figure 5:**
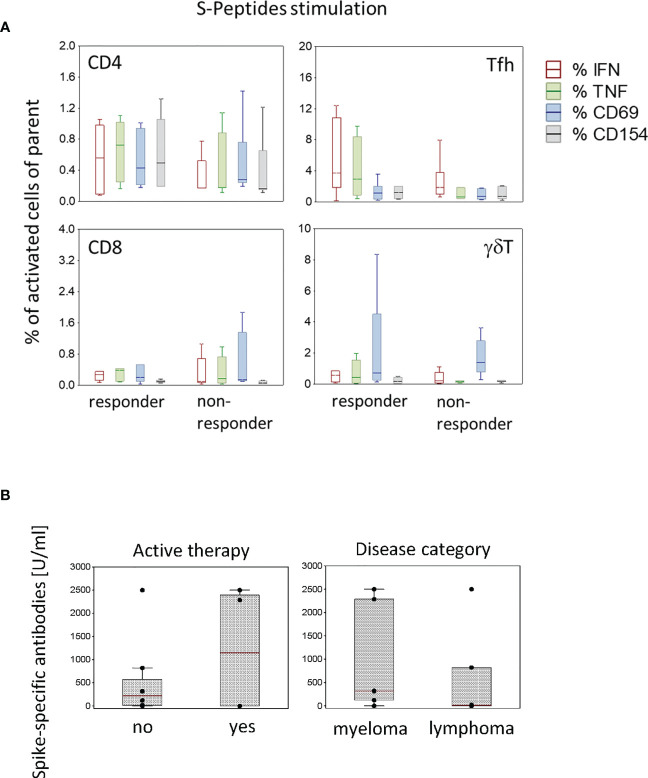
Activated T cell subtypes in antibody responder (N=6) and non-responder (N=6) patients **(A)** and spike-specific antibody concentrations in patients (N=12) according to active therapy (no, N=8; yes, N=4) and disease category (myeloma, N=6; lymphoma N=6) **(B)** after two vaccine doses. Box plots with median, interquartile range and minimum-to-maximum whiskers show the percentages of activated T cell subtypes **(A)** and the spike-specific antibody concentrations **(B)**. Data analysis with Mann-Whitney U Test, no significant differences were measured.

In this study, SARS-CoV-2-specific antibody concentrations in the blood were measured only in patients. The time course of spike-specific antibody concentrations is shown in **Table 2**. About four weeks after the second dose of the vaccine (**Table 2**, time point T3), three patients developed a strong antibody response (819.4, 2287 and 2500 U/ml), three a moderate response (124, 313 and 323 U/ml) and six almost no specific antibody response (≤ 23.8 U/ml). According to the antibody concentration about four weeks after the second vaccine dose (**Table 2**, time point T3), patients were categorized in responders (> 23.8 U/ml, n=6) and non-responders (≤ 23.8 U/ml, n=6). These two groups did not differ regarding the frequency of SARS-CoV-2-specific T cells (**Figure 5A**). In responders, the antibody concentrations declined after 9 to 12 weeks (**Table 2**). Neither an active therapy in the month before vaccination nor the disease category was associated with the concentrations of SARS-CoV-2-specific antibodies at that time point in these patients (**Figure 5B**).

**Table 2:** Concentration (U/ml) of antibodies against SARS-CoV-2 spike antigens in cancer patients (N=12). Time points (T) between analyses range from 3 to 4 weeks.

	T1	T2 Second vaccine	T3	T4	T5	T6	T7	T8	T9
Pat_1	N/A*	0	323	200	N/A	N/A	N/A	N/A	N/A
Pat_2	0	0	23.8	6.77	0	0	0	N/A	N/A
Pat_3	0	0	819.4	514.8	315.3	204	126	N/A	N/A
Pat_4	0	0	0	0	0	0	0	10	N/A
Pat_5	0	N/A	313	0	164	187	155	163	109
Pat_6	0	0	2	14	84	73	73	64	N/A
Pat_7	0	31	2287	1516	1131	1065	974	726	N/A
Pat_8	0	0	124	145	131	N/A	51	22	0
Pat_9	N/A	N/A	0	0	0	N/A	N/A	N/A	N/A
Pat_10	N/A	0	0	0	0	N/A	N/A	N/A	N/A
Pat_11	N/A	0	0	9	35	N/A	N/A	N/A	N/A
Pat_12	0	11	2500	1481	562	255	N/A	N/A	N/A

*N/A, not analyzed.

## Discussion

### Distribution of SARS-CoV-2-specific T cell subtypes in patients versus controls

First of all, after SARS-CoV-2 vaccination, both patients and controls developed a proper detectable cellular immune response against the viral spike peptides. This is in line with other publications showing that COVID-19 mRNA vaccines elicit a strong and robust immune response to the viral spike proteins (4, 8, 9). In our patients, the cellular response was even higher for certain T cell subtypes compared to controls. Tfh and, in part, γ δT cells were the main responder T cells in patients. Whereas in patients the distribution of analysed T cell subtypes was not related to sex, in controls male subjects had a lower percentage of SARS-CoV-2-specific CD154+ γδT cells. Thus, the comparison of these T cell subtypes between patients and controls might be biased by sex distribution and needs to be interpreted with caution. Tfh cells represent a key cell subtype important for the generation of antigen-specific humoral immune responses (10). Dependent upon signals provided by CD4+ Tfh cells, including cytokines such as IL-4 and IFN-γ, which promote B cell isotype switching, activated B cells undergo affinity maturation and differentiation in the germinal center (11). A part of Tfh cells are in the peripheral blood (10% of the CD4+ T cell compartment) and, similar to the lymphoid tissue Tfh cells, they express the chemokine receptor CXCR5 (12). One explanation for the higher frequency of activated Tfh cells in patients might be given by the fact that Tfh cells per se are altered in patients with hematologic malignancies. In chronic lymphocytic leukemia patients, Tfh cells are expanded and phenotypically distinct (13). They express higher levels of the Tfh-associated activation markers PD-1 and ICOS and are skewed to an IFN-γ Type 1 phenotype (13). Compared to controls, in our study Tfh cells of patients seem to have a higher reactivity to specific antigens such as S and CEF-Peptides, at least the IFN- and TNF-producing Tfh cells. The basal level, i.e., the frequencies of IFN- and TNF-producing Tfh cells in the unstimulated samples, was also higher in patients. Thus, *a priori* patients with hematologic malignancies had a higher frequency of pre-activated Tfh cells, which, besides other functional characteristics of these cells in patients, might have led to the higher Tfh cell response to S and CEF-Peptides. In conclusion, although a higher number of distinct Tfh cells might be unfavorable for the course of the hematological malignancy, it might promote a better response to vaccine-specific antigens. In patients, compared to controls, we also detected a higher frequency of S-Peptides-specific CD4+IFN+ cells and CEF-Peptides-specific CD4+INF+ and CD4+TNF+ cells. By using a similar method to identify SARS-CoV-2-specific T cells, Rouhani et al. (8) found equal levels of CD4+ and CD8+ antigen-specific T cells in cancer patients and controls. This discrepancy might be explained in part by the type of cancer: Whereas in our study, participating patients had hematological malignancies only, the majority of patients in the study of Rouhani et al. had solid cancers. Of note, a higher abundance of γδT cells was observed in patients. Many reports show that γδT cells play an important role in the generation of anti-tumor immune responses (14, 15). Furthermore, these lymphocytes stimulate the maturation of dendritic cells (16, 17), thereby facilitating a proper antigen presentation to conventional T cells. The unbiased t-SNE plot analysis in our study revealed that in patients, but not in controls, TCRγδ was expressed in parts of the Tfh cell compartment, indicating the presence of TCRγδ+CXCR5+ cells. In this regard, it has been published that in mice, γδT cells can rapidly induce CXCR5 expression after immunization (18). These TCRγδ+CXCR5+ cells function as antigen-presenting cells to CD4 T cells, inducing the differentiation of Tfh cells and thereby controlling the humoral immune response (18). Concerning the activation state of γδT cells in our study, the t-SNE analysis, revealed partially higher numbers of IFN and CD69 expressing γδT cells in patients in S-Peptides-stimulated samples. Thus together, indirectly by their immune stimulatory properties, the higher abundance of γδT cells in patients might facilitate the better responsiveness to the vaccine, mirrored by the higher frequency of S-Peptides-specific T cells.

Concerning the general T cell activation capacity, we found that γδT cells were less responsive to CD3/CD28 stimulation in patients. It is noteworthy that γδT cells per se have a diminished responsiveness to standard T-cell receptor stimulation *via* CD3/CD28 (19). They display regulatory/immunosuppressive activity on αβT cells when co-cultured with CD3/CD28 mAB (20, 21). Although we did not assess markers for cell exhaustion, it might be speculated that γδT cells in patients have an altered functionality and less responsiveness to CD3/CD28 stimulation due to the tumor microenvironment and chronic stimulation with tumor antigens. This might also be one explanation for the lower frequency of CD8+CD69+ T cells in response to CD3/CD28 stimulation observed in patients compared to controls. However, despite this general reduced responsiveness, the ability to develop a newly induced antigen-specific immune response in patients was comparable to controls and particularly even stronger as seen for CD4+ T and Tfh cells.

### Correlation between the frequency of the particularly activated T cell subtypes

To verify whether a cancer- and immunosuppressed-experienced immune system as that of hematologic malignancy patients is still capable of responding to a specific antigen, we used the CEF peptide pool for immune cell stimulation. This peptide mix includes sequences of common viruses that anyone has been in contact with once in life (i.e., human cytomegalovirus, Epstein-Barr virus and influenza A virus). In a healthy immune system, a cellular immune response specific to these peptides is expected but might be altered in cancer patients. Similarly, the cellular response to SARS-CoV-2 antigens might be affected in these patients. However, in our study this was not the case. We observed a strong correlation between these antigen-specific immune responses in both patients and controls. This is of special importance for patients where a proper response to recall antigens such as CEF-Peptides reflects the ability to generate antigenic immune responses and thus might predict the capacity to generate an anti-SARS-CoV-2 response, either to infection or to vaccination. In this regard, we suggest that especially in vulnerable patients a pre-validation of immune responses with recall antigens would indicate the ability to respond to vaccination. In case of no response to recall antigens, other measures have to be taken into consideration for the safety of these patients against infection. Again, the focus here should be on Tfh and γδT cells, at least in blood cancer patients as they show a very strong correlation between the frequency of recall antigen-specific T cells and the currently generated SARS-CoV-2-specific cells in these T cell compartments. The negative association between the frequency of antigen-specific activated T cells and the frequency of CD3/CD28 activated was similar in patients and controls. This negative association might reflect a general exhaustion of T cells during viral infection or, in this case, through immune challenging after vaccination. However, no exhaustion markers have been measured in our study. Thus, this assumption needs validation in future studies.

### Active therapy before the vaccination period impacts T cell responses in patients

Patients with an active therapy within one month before vaccination developed a stronger immune response to spike proteins of the virus compared to patients without treatment in this period. The frequency of CD4+ and Tfh cells producing IFN or TNF or expressing CD69 and CD154 were significantly higher in these patients, as seen by means of S-Peptides stimulation (**Figure 4**). T-SNE analysis revealed a higher abundance of γδT cells in patients without active therapy in this period. Still, T cells expressing IFN or CD69 were more frequent in patients with an active therapy. The therapy of the four patients treated before the vaccination period included ibrutinib, lenalidomide, daratumumab-lenalidomide-dexamethasone and bortezomib. The nature of this treatment might explain in part the higher frequency of SARS-CoV-2-specific T cells in treated patients compared to untreated patients and even controls. It has been shown that ibrutinib (a Bruton’s tyrosine kinase inhibitor) therapy in chronic lymphocytic leukemia patients restores the TCR repertoire diversity and alters the T cell subtype composition by exerting a Th1 selective pressure (22, 23). Thus, in patients with this treatment, CD4+ T cells might be prone to a higher reactivity to new antigens such as SARS-CoV-2 peptides. By now, no data is available regarding the administration of ibrutinib and the impact on Tfh cells. Since Tfh are CD4+ derived T cells, it is assumable that ibrutinib has similar modulatory effects also on this T cell subtype. In sum, ibrutinib has assigned an immunomodulatory capacity with a higher T cell proliferative ability, degranulation and cytokine secretion (24, 25) which might be favorable not only in terms of cancer therapy but also for the generation of antigen-specific immune responses. Another common therapy in hematologic cancer patients (i.e., multiple myeloma) is the treatment with immunomodulatory drugs (IMiDS) such as lenalidomide. These drugs clearly impact the activity of immune cells and also act directly on myeloma cells, thereby altering their proliferative capacity, as reviewed in (26). *In vitro* studies have shown that treatments with IMiDS-enhanced T cell proliferation, IL-2 and IFN-γ secretion and NK and NKT cell activation (27, 28). In particular, IMiDS enhance DC antigen presentation, thereby leading to activation of CD4+ and CD8+ T cells and increased IFN-γ production (29, 30). Thus, especially for an antigen-specific driven immune response, as expected after SARS-CoV-2 vaccination, this presumably is important and might partially explain the higher frequency of SARS-CoV-2-specific CD4+ T cells seen in treated patients in our study. Enhanced antigen presentation might also be of relevance for the response to recall antigens as we observed a higher CD8+ T cell response to CEF-Peptides in treated patients. Bortezomib, a proteasome inhibitor used in treating multiple myeloma, also has immunomodulatory properties. Among others, it enhances the production of IFN and effector molecules such as perforin and granzyme B and downregulates PD-1 expression in CD8+ T cells (31, 32). Taken together, the mentioned cancer therapies classically directed against tumor growth additionally have immunostimulatory effects, which might not only be beneficial in terms of tumor immunosurveillance, but also for the generation of antigen-driven immune responses, as is the case regarding the current vaccine.

### Disease category and cellular immune responses

Another point of interest in our study was to find out whether patients with different hematologic malignancies show differences in their ability to establish a SARS-CoV-2-specific immune response after vaccination. In both analyzed categories, myeloma and lymphoma, a dysfunction of immune responses to newly acquired foreign antigens might be expected due to disease-related alterations at the immune system level. However, we observed a higher frequency of SARS-CoV-2-specific Tfh cells in myeloma patients compared to lymphoma patients. Reported immune alterations in multiple myeloma include defects in T cell function, a reduction of peripheral CD4+ and CD8+ T cells, abnormal Th1/Th2 ratio, a decrease in CD4/CD8 T cell ratio and a reduction in NKT cells (33). No special changes at the Tfh cell level have been reported by now. In lymphoma patients (e.g., chronic lymphocytic leukemia, CLL), an increased number of circulating Tfh cells is shown (34, 35). At the same time, the leukemic B cells in these patients have a regulatory phenotype playing a central role in driving immunosuppression and progressively inhibiting immune responses. Among others, by secreting IL-10 they inhibit CD8+ cytotoxic and CD4+ activated effector cells (36). Besides the impairment of T cell activity, leukemic B cells promote the expansion and functionality of Treg, which contribute to suppressing specific immune responses (37–39). Thus, although lymphoma patients have a high frequency of circulating Tfh cells, the ability of these cells to respond to new antigens such as SARS-CoV-2 might be reduced compared to myeloma patients, due to the lymphoma patients’ immunosuppressive B cells. In contrast, T cells of myeloma patients were less responsive to CD3/CD28 stimulation compared to lymphoma patients. Although only speculative, this might be a sign of exhaustion. In this regard, Zelle-Rieser et al. demonstrated that in myeloma patients, T cells at the tumor site within the bone marrow are immune-suppressed, largely exhausted and senescent (40). Notably, compared to lymphoma patients, a higher abundance of γδT cells in myeloma patients with t-SNE analysis was identified in the compartments of CD4, CD8 and Tfh cells. Also, the frequency of IFN-producing or CD69-expressing γδT cells was higher in myeloma patients. Thus, as mentioned above, the immunomodulatory properties of γδT cells on Tfh cells might partly explain the higher frequency of S-Peptides-specific Tfh cells detected in myeloma patients after vaccination.

### SARS-CoV-2-specific antibodies and specific cellular immune response in patients

Considering the antibody concentrations at 3-4 weeks after the second vaccination dose, patients were classified as responders and non-responders. All activated T cell subtypes were similarly distributed within the two categories, indicating no relationship between the developed cellular immune response and antibody concentrations after vaccination. This result is in line with the findings of other groups analyzing T cell and antibody responses in cancer patients after SARS-CoV-2 vaccination (8, 41–43). In contrast, in healthy vaccinated individuals especially the frequency of Tfh cells directed to viral spike peptides correlated with the concentration of specific antibodies (44). Furthermore, in convalescents as well as in vaccinated individuals Tfh cells correlated with spike-specific IgG and memory B cell responses for several months (44, 45). In particular, here the CXCR3+ but not the CXCR3- phenotype correlated with the antibody concentrations. However, we could not verify these findings in our controls, and in patients the frequency of activated S-Peptides-specific Tfh cells was not associated with the specific antibody concentrations when we compared responders with non-responders. One explanation for this difference might be in the way Tfh cells have been characterized. Whereas in the mentioned publications CXCR3 was taken into consideration to distinguish between Type 1 and Type 2 Tfh cells, in our study this marker was not measured. In conclusion, for future vaccination studies it is important to identify the certain Tfh cell subtypes.

We observed no significant difference in antibody concentrations when comparing patients on active cancer therapy with patients who were not. This is in line with the findings of other groups (3, 42) where an active cancer therapy did not include B cell depletion. Also, the type of malignancy was not significantly associated with SARS-CoV-2 spike-specific antibodies. However, although both lymphoma and myeloma are per se accompanied by impaired T and B cell functionality, the generation of antigen-specific immune responses seems not to be affected at the T cell level. As mentioned above, the patient’s therapy presumably has an impact on this specific immune response. At least for ibrutinib, lenalidomide, and bortezomib, immune-stimulatory properties are reported. While at the cellular level we clearly detected a higher immune response to SARS-CoV-2 antigens in patients with these therapies, at the humoral level this was visible only in trend. However, out of the 12 patients, four did not develop an antibody response after vaccination, corresponding to a seroconversion rate of 67%. This is in a similar range as observed in other studies in which the seroconversion rates in hematologic malignancies were lower compared to solid cancer types or controls (2, 3, 8, 42). However, all four non-responders, similar to the responders, developed a robust SARS-CoV-2 T cell answer after vaccination. This finding is consistent with other works showing specific T cell responses in patients without seroconversion after vaccination (8, 42). Missing antibody responses might be impacted by B cell-depleting therapies (anti-CD20 treatment). For example, for a proper humoral response, it has been shown that the optimal interval for SARS-CoV-2 vaccination after the final dose of anti-CD20 treatment was 5.5 months, reviewed in (46). Two patients in our group received Rituximab treatment after vaccination and did not develop an antibody response, pointing out that the interval was too short for B cell recovery. However, also these patients developed a proper T cell response, sustaining similar findings in lymphoma patients (47). These and our data point out that the control of vaccine efficacy should include measurements at the cellular level besides serology, at least in vulnerable patients. A proper T cell response to SARS-CoV-2 antigens might build the first line of defense against this virus, reducing the viral load in a putative infection and thereby partially overcoming the lack of specific antibodies in non-responders.

### Strengths and limitations

Our study presents a detailed analysis of the cellular immune response in patients with hematologic malignancies and matched controls after SARS-CoV-2 vaccination. To our knowledge, for the first time the frequency and function of certain SARS-CoV-2-reactive T cell subtypes (e.g., CD4, CD8, Tfh and γδT) are analyzed in myeloma and lymphoma patients. Our results point out that in hematologic malignancies CD4+ and Tfh cells play the major role in the immune response to the SARS-CoV-2 vaccine. Furthermore, γδT cells indirectly seem to support the generation of the antigen-specific immune response, due to their immunomodulatory capacity. Our study also shows that particularly immunomodulatory therapies in cancer patients should be continued during the vaccination period as they may increase the cellular immune response to the vaccine. However, a limitation of our study is the low number of study participants (12 patients and 12 controls) which does not allow the performance of several stratified analyses as, for example, for sex distribution combined with cancer types and certain therapies. A more complex statistical analysis *via* adjusted models, accounting for several confounding factors, was likewise not possible in these small participant groups. Therefore, the data has to be interpreted with caution. To better entangle the relationship between cancer types, therapy and vaccine efficacy at cellular and humoral levels, these analyses have to be validated in larger patient cohorts. Another limitation of our study is that serology has not been performed in controls, which does not allow to analyze the relationship between the cellular and humoral immune response in healthy individuals. It is also limiting that blood samples before vaccination could not be collected for the analysis of the pre-vaccination immune responses.

## Conclusion

Overall, our findings suggest that myeloma patients and lymphoma patients are capable of generating a robust anti-SARS-CoV-2 cellular immune response after vaccination and that the type of active therapy before and during the vaccination period is important. Furthermore, the finding that certain types of immunomodulatory therapies used to improve the course of hematologic malignancies are beneficial for generating an antigen-driven immune response might be of relevance also for other cancer entities.

## Data availability statement

The original contributions presented in the study are included in the article/**Supplementary Material**. Further inquiries can be directed to the corresponding author.

## Ethics statement

The studies involving human participants were reviewed and approved by Ethics Committee of the Medical Association Saxony Anhalt (#16/21). The patients/participants provided their written informed consent to participate in this study.

## Author contributions

RP, SF, GH conceived and designed the present investigation. RP, SW, SF, processed and stored patients and controls samples. AP, GH, developed and performed multi-parameter flow cytometric analysis. AS, SW, performed antibody measurements. CZ helped with study protocol and ethic application. AZ, SF organized funding, acquisition and resources. GB helped with supervision of clinical investigation, AM did the tSNE analysis, GH did the data analysis and drafted the manuscript. All authors contributed to the article and approved the submitted version.
