# Supplementary material for: Characterization of post-vaccination SARS-CoV-2 T cell subtypes in patients with different hematologic malignancies and treatments
Source: Front Immunol. 2023 Apr 28;14:1087996. doi: 10.3389/fimmu.2023.1087996 (PMC10177659; doi:10.3389/fimmu.2023.1087996)
Supplement: Supplementary file 1 [file DataSheet_1.zip › Supplementary_Material_SARS_CoV_2 T cells.docx]

**Supplementary Material**

**Supplementary Tables**

**Table S1.** Antibodies used for the immune cell staining in the flow cytometric analysis

| **SPECIFICITY** | **FLUOROCHROME** | **CLONE** | **CATALOG #** | **VENDOR** | **Titer (µl/test)** | **Coctail** |
| --- | --- | --- | --- | --- | --- | --- |
| TCRγδ | PE | B1 | 331430 | BioLegend | 0.5 | A |
| CXCR5 | BV711 | J252D4 | 356934 | BioLegend | 0.5 | A |
| CD3 | Spark Blue 550 | SK7 | 344852 | BioLegend | 0.5 | B |
| CD4 | PerCP | SK3 | 344624 | BioLegend | 1 | B |
| CD8a | APC-eF780 | RPA-T8 | 47-0088-42 | ThermoFisher | 0.25 | B |
| CD19 | APC/Fire810 | HIB19 | 302272 | BioLegend | 0.25 | B |
| CD56 | BV510 | HCD56 | 318340 | BioLegend | 0.5 | B |
| IFN-gamma | Pacific Blue | 4S.B3 | 502522 | BioLegend | 0.25 | C |
| TNF | BV605 | MAb11 | 502909 | BioLegend | 0.25 | C |
| CD69 | FITC | FN50 | 310904 | BioLegend | 0.5 | C |
| CD154 | PE-Dazzle594 | 24-31 | 310840 | BioLegend | 0.25 | C |

**Table S2.** T cell responses after second SARS-CoV-2 vaccination in cancer patients (N=12). Data of the flow cytometric analysis of PBMCs stimulated with S-Peptides, CEF-Peptides, CD3/CD28 or left unstimulated. Shown is the median with IQR of the percentage of activated cells in the certain parent T cell subtype.

| % activated T cell subtypes | | | | | | |
| --- | --- | --- | --- | --- | --- | --- |
|  |  | **% IFN** |  | **% TNF** | **% CD69** | **% CD154** |
| Stimulus |  |  |  |  |  |  |
| S-Peptides* |  |  |  |  |  |  |
| CD4 |  | 0.36 (0.17-0.80) |  | 0.50 (0.18-0.95) | 0.36 (0.23-0.85) | 0.39 (0.18-1.03) |
| CD8 |  | 0.25 (0.08-0.52) |  | 0.27 (0.09-0.58) | 0.18 (0.10-0.94) | 0.09 (0.05-0.13) |
| Tfh ^§^ |  | 3.39 (1.41-5.92) |  | 2.12 (0.55-4.14) | 0.95 (0.33-1.75) | 1.04 (0.41-2.00) |
| γδT ^#^ |  | 0.39 (0.09-0.81) |  | 0.19 (0.08-0.50) | 1.08 (0.37-3.20) | 0.18 (0.06-0.38) |
| CD3/CD28 |  |  |  |  |  |  |
| CD4 |  | 1.06 (0.35-2.60) |  | 3.99 (2.02-15.27) | 20.33 (12.12-39.22) | 17.47 (11.34-39.34) |
| CD8 |  | 1.82 (0.89-7.82) |  | 1.75 (1.01-7.37) | 10.88 (5.69-29.96) | 1.75 (0.22-2.50) |
| Tfh |  | 2.02 (1.32-8.41) |  | 5.09 (3.58-15.56) | 20.88 (15.18-44.65) | 22.74 (18.56-47.65) |
| γδT |  | 1.01 (0.09-1.94) |  | 0.43 (0.19-2.86) | 4.34 (0.55-20.00) | 0.97 (0.28-1.33) |
| CEF-Peptides^γ^ |  |  |  |  |  |  |
| CD4 |  | 0.15 (0.07-0.44) |  | 0.16 (0.03-0.32) | 0.18 (0.11-0.35) | 0.07 (0.05-0.18) |
| CD8 |  | 0.62 (0.05-1.59) |  | 0.62 (0.04-1.49) | 0.15 (0.09-1.53) | 0.04 (0.02-0.06) |
| Tfh |  | 1.10 (0.32-2.08) |  | 0.79 (0.07-1.97) | 0.90 (0.19-1.91) | 0.44 (0.24-0.79) |
| γδT |  | 0.32 (0.07-1.67) |  | 0.09 (0.05-0.45) | 0.69 (0.39-1.50) | 0.25 (0.07-0.33) |
| Unstimulated |  |  |  |  |  |  |
| CD4 |  | 0.05 (0.02-0.08) |  | 0.05 (0.03-0.07) | 0.12 (0.07-0.15) | 0.13 (0.06-0.15) |
| CD8 |  | 0.02 (0.01-0.02) |  | 0.02 (0.01-0.04) | 0.09 (0.06-0.15) | 0.04 (0.02-0.05) |
| Tfh |  | 0.57 (0.26-1.06) |  | 0.36 (0.12-0.64) | 0.28 (0.15-0.63) | 0.45 (0.25-0.98) |
| γδT |  | 0.26 (0.06-0.54) |  | 0.10 (0.06-0.25) | 0.97 (0.23-2.38) | 0.12 (0.06-0.18) |

* S: pool of peptides covering the whole spike-protein of SARS-CoV-2

**^γ^** CEF: pool of peptides from human Cytomegalovirus, Epstein-Barr virus and Influenza A virus

§ Tfh: T follicular helper cells

# γδT: gamma delta T cells

**Table S3.** T cell responses after second SARS-CoV-2 vaccination in controls (N=12). Data of the flow cytometric analysis of PBMCs stimulated with S-Peptides, CEF-Peptides, CD3/CD28 or left unstimulated. Shown is the median with IQR of the percentage of activated cells in the certain parent T cell subtype.

| % activated T cell subtypes | | | | | | |
| --- | --- | --- | --- | --- | --- | --- |
|  |  | **% IFN** |  | **% TNF** | **% CD69** | **% CD154** |
| Stimulus |  |  |  |  |  |  |
| S-Peptides^*^ |  |  |  |  |  |  |
| CD4 |  | 0.16 (0.09-0.22) |  | 0.26 (0.18-0.36) | 0.39 (0.34-0.44) | 0.40 (0.32-0.47) |
| CD8 |  | 0.18 (0.13-0.40) |  | 0.18 (0.14-0.60) | 0.57 (0.35-1.08) | 0.14 (0.09-0.16) |
| Tfh |  | 0.85 (0.39-0.99) |  | 0.63 (0.35-0.85) | 0.51 (0.38-0.78) | 0.75 (0.52-1.05) |
| γδT |  | 0.19 (0.13-0.52) |  | 0.32 (0.15-0.55) | 1.09 (0.72-1.69) | 0.21 (0.10-0.41) |
| CD3/CD28 |  |  |  |  |  |  |
| CD4 |  | 1.01 (0.58-1.60) |  | 4.78 (4.16-6.30) | 29.36 (18.26-43.12) | 28.94 (16.23-36.72) |
| CD8 |  | 3.07 (1.82-6.61) |  | 3.79 (2.52-6.68) | 21.72 (18.01-35.28) | 2.12 (1.23-6.01) |
| Tfh |  | 2.24 (1.512.61) |  | 9.04 (5.40-10.84) | 33.55 (23.41-43.23) | 36.70 (18.96-46.10) |
| γδT |  | 3.15 (1.21-5.39) |  | 4.32 (3.23-5.30) | 18.25 (11.79-21.08) | 2.01 (1.40-2.89) |
| CEF-Peptides^γ^ |  |  |  |  |  |  |
| CD4 |  | 0.04 (0.02-0.07) |  | 0.03 (0.02-0.05) | 0.14 (0.10-0.20) | 0.10 (0.08-0.14) |
| CD8 |  | 0.26 (0.12-0.63) |  | 0.23 (0.11-0.53) | 0.53 (0.22-1.33) | 0.04 (0.03-0.06) |
| Tfh |  | 0.18 (0.13-0.36) |  | 0.08 (0.06-0.17) | 0.23 (0.14-0.34) | 0.42 (0.23-0.59) |
| γδT |  | 0.14 (0.05-0.33) |  | 0.12 (0.09-0.28) | 0.69 (0.35-1.57) | 0.13 (0.08-0.40) |
| Unstimulated |  |  |  |  |  |  |
| CD4 |  | 0.03 (0.02-0.05) |  | 0.02 (0.02-0.04) | 0.12 (0.11-0.14) | 0.10 (0.07-0.14) |
| CD8 |  | 0.02 (0.01-0.04) |  | 0.01 (0.01-0.03) | 0.19 (0.13-0.34) | 0.04 (0.02-0.05) |
| Tfh^§^ |  | 0.27 (0.11-0.38) |  | 0.08 (0.05-0.18) | 0.22 (0.14-0.31) | 0.39 (0.29-0.56) |
| γδT^#^ |  | 0.10 (0.07-0.35) |  | 0.13 (0.06-0.27) | 0.72 (0.48-1.35) | 0.24 (0.11-0.39) |

* S: pool of peptides covering the whole spike-protein of SARS-CoV-2

**^γ^** CEF: pool of peptides from human Cytomegalovirus, Epstein-Barr virus and Influenza A virus

§ Tfh: T follicular helper cells

# γδT: gamma delta T cells

**Table S4.** Detailed characteristics of patients

| **Patient** | **Age** | **Sex** | **Type of cancer^µ^** | **Diagnosis date** | **Disease status at vaccination** | **Active therapy**^#^ | **Last cancer therapy**^§^ | **First vaccine** | **Second vaccine** | **Vaccine Type** | **Blood for T cell analysis** |
| --- | --- | --- | --- | --- | --- | --- | --- | --- | --- | --- | --- |
| 1 | 55 | male | MM | Mar-21 | Progress | no | Daratumumab-chemotherapy-Bortezomib | 03/09/21 | 06/01/21 | AZD1222 | 07/14/21 |
| 2 | 63 | female | FCL | Jan-11 | Progress | no | Rituximab-chemotherapy | 01/28/21 | 02/18/21 | BNT162b2 | 09/24/21 |
| 3 | 61 | male | CLL | Jul-03 | Remission (clinical) | no | Ibrutinib (mono) | 01/28/21 | 02/18/21 | BNT162b2 | 05/19/21 |
| 4 | 60 | male | MCL | Oct-16 | Remission (clinical) | Ibrutinib (mono) | Ibrutinib (mono) | 01/28/21 | 02/18/21 | BNT162b2 | 05/19/21 |
| 5 | 76 | female | MM | Nov-19 | Remission (VGPR*) | no | Chemotherapy-Bortezomib | 01/28/21 | 02/18/21 | BNT162b2 | 05/19/21 |
| 6 | 47 | male | FCL | Feb-16 | Progress | no | no | 01/28/21 | 02/18/21 | BNT162b2 | 03/17/21 |
| 7 | 44 | male | MM | Sep-19 | Remission (VGPR) | Lenalidomide | Lenalidomide | 01/28/21 | 02/18/21 | BNT162b2 | 05/19/21 |
| 8 | 58 | male | MM | Jul-20 | Remission (VGPR) | no | Chemotherapy-Bortezomib | 01/28/21 | 02/18/21 | BNT162b2 | 03/18/21 |
| 9 | 70 | male | CLL | Oct-13 | Progress | no | Rituximab-Venetoclax | 02/05/21 | 02/26/21 | BNT162b2 | 04/22/21 |
| 10 | 90 | female | FCL | Dec-20 | Stable disease | no | no | 03/18/21 | 04/09/21 | BNT162b2 | 05/11/21 |
| 11 | 65 | female | MM | Aug-19 | Remission | Daratumumab-Lenalidomide-Dexamethasone | Daratumumab-Lenalidomide-Dexamethasone | 04/08/21 | 04/28/21 | BNT162b2 | 05/12/21 |
| 12 | 72 | female | MM | Nov-19 | Progress | Bortezomib | Bortezomib | 04/08/21 | 05/04/21 | BNT162b2 | 05/19/21 |

^µ^Type of cancer: Follicular Lymphoma (FCL), Chronic Lymphocytic Leukemia (CLL), Mantle Cell Lymphoma (MCL)

*VGPR=very good partial remission according to IMWG-criteria

^#^Active therapy means therapy before and during the vaccination period. Therapy started a minimum one month before 1st vaccination and ended more than one month after 2nd vaccination.

^§^Last therapy includes patient therapies more than one month before the 1st dose and starting after the 2nd vaccine dose and, in one patient, after the 1st dose.

**Table S5.** Detailed characteristics of controls

| **Control** | **Age** | **Sex** | **Second vaccine** | **Vaccine Type** | **Blood for T cell analysis** |
| --- | --- | --- | --- | --- | --- |
| 1 | 70 | male | 02/18/21 | BNT162b2 | 05/10/21 |
| 2 | 89 | female | 01/25/21 | BNT162b2 | 05/10/21 |
| 3 | 76 | female | 02/18/21 | BNT162b2 | 05/10/21 |
| 4 | 63 | female | 02/18/21 | BNT162b2 | 05/10/21 |
| 5 | 58 | male | 02/25/21 | BNT162b2 | 05/10/21 |
| 6 | 60 | male | 02/18/21 | BNT162b2 | 05/10/21 |
| 7 | 55 | male | 02/10/21 | BNT162b2 | 05/10/21 |
| 8 | 67 | female | 01/25/21 | BNT162b2 | 05/10/21 |
| 9 | 64 | female | 01/21/21 | BNT162b2 | 05/10/21 |
| 10 | 57 | male | 01/25/21 | BNT162b2 | 05/10/21 |
| 11 | 52 | female | 05/23/21 | BNT162b2 | 06/17/21 |
| 12 | 55 | female | 07/18/21 | BNT162b2 | 09/13/21 |

**Supplementary Results**

*Relation of disease state, age and sex to T cell responses in patients*

Concerning the disease state at the time of vaccination, patients were categorized into the groups remission/stable disease (including clinical remission, very good partial remission according to IMWG-criteria and stable disease, N=7) and disease progression (with progressive disease, N=5). Neither in unstimulated nor in the particularly stimulated samples a difference between these two patients’ groups was observed in the analyzed immune responses (Supplementary Table S10).

Of note, in patients the distribution of the analyzed T cell subtypes was not related to age or sex (Supplementary Table S6 and Table S8).

*Relation of age and sex to T cell response in controls*

In controls, three T cell subtypes were related to age: S-Peptides-specific CD4+IFN+ and Tfh+TNF+ cells were negatively whereas CEF-Peptides-specific Tfh+CD154+ were positively associated to age (Supplementary Table S7). Female compared to male controls, had a higher percentage of S-Peptides-specific γδT+CD154+ cells and a lower percentage of unstimulated γδT+IFN+ cells (Supplementary Table S9).

*Relation of time interval past after second vaccine dose and T cell responses*

Another factor impacting the magnitude of T cell responses developed after vaccination might be the number of days past after the second vaccine dose and collection of blood for T cell analyses (Table 1). We present these relationships in Table S11 (patients) and Table S12 (controls) as Excel files in Supplementary Tables S6-S12. The Spearman rank correlation revealed that in controls there was no association between the number of days past after the second vaccine and the percentage of specifically activated T cell subtypes. In patients, only one association was observed, namely that a higher number of days after the second vaccine correlated with a higher percentage of CD8+CD154+-S-Peptides activated T cells (R=0.65, P=0.02).

**Supplementary Figure legends**

**Figure S1**: Analysis of activated T cells after two vaccine doses. Exemplary dot plots of CEF-Peptides (**A**) and CD3/CD28 stimulated samples (**B**) from one control subject. T cells specific for CEF-Peptides were identified after 6h stimulation of PBMC with a pool of peptides from Cytomegalovirus, Epstein-Barr virus and Influenza A virus (CEF-Peptides). Cells of interest (CD4, CD8, Tfh, γδT) were gated as shown in Figure 1. Intracellular staining of cytokines and activation markers followed by flow cytometry served as the read-out assay. Activated T cell subtypes (CD4, CD8, Tfh, γδT) specific for CEF or activated by CD3/CD28 were identified by the production of IFN and TNF or the expression of the activation markers CD69 or CD154.

**Figure S2**: Activated T cell subtypes in unstimulated and CEF-Peptides stimulated samples of patients (N=12#) and controls (N=12) after two vaccine doses (**A**). Box plots with median, interquartile range and minimum-to-maximum whiskers show activated T cell subtypes as a percentage of the parent population (CD4, Tfh, CD8, γδT). Cells were identified after 6h stimulation of PBMC and intracellular staining of cytokines (IFN, TNF) and activation markers (CD69, CD154) followed by flow cytometry. Data analysis with Mann-Whitney U Test, *p<0.05, **p<0.005. Please note the different scaling for better visibility. # Due to the low blood amount, the CEF-Peptides stimulation analysis was not performed in one patient. (**B**) Correlation between the percentages of the particularly activated T cell subtypes shown separately for patients and controls. Activated T cell subtypes are the percentage of parent population (CD4, Tfh, CD8, γδT) producing cytokines (IFN, TNF) or activation markers (CD69, CD154) after 6h stimulation with S-Peptides, CD3/CD28 or CEF-Peptides. Heat map is presenting the correlation coefficients (R) gained from the Spearman Rank correlation test, red and blue colors indicate positive and negative correlations, respectively, *p<0.05, **p<0.005, ***p<0.0005.

**Figure S3**: Activated T cell subtypes in unstimulated or CEF-Peptides stimulated samples of patients regarding (**A**) active therapy (no, N=8#, yes N=4) and (**B**) disease category (myeloma, N=6#, lymphoma, N=6) after two vaccine doses. Box plots with median, interquartile range and minimum-to-maximum whiskers show activated T cell subtypes as a percentage of the parent population (CD4, Tfh, CD8, γδT). Cells were identified after 6h stimulation of PBMC and intracellular staining of cytokines (IFN, TNF) and activation markers (CD69, CD154) followed by flow cytometry. Active therapy means therapy before and during the vaccination period. Therapy started a minimum one month before 1st vaccination and ended more than one month after 2nd vaccination. Data analysis with Mann-Whitney U Test, *p<0.05, **p<0.005. Please note the different scaling for better visibility. # Due to the low blood amount, the CEF-Peptides stimulation analysis was not performed in one patient.
