# Supplementary figures and images for: Characterization of post-vaccination SARS-CoV-2 T cell subtypes in patients with different hematologic malignancies and treatments
Source: Front Immunol. 2023 Apr 28;14:1087996. doi: 10.3389/fimmu.2023.1087996 (PMC10177659; doi:10.3389/fimmu.2023.1087996)

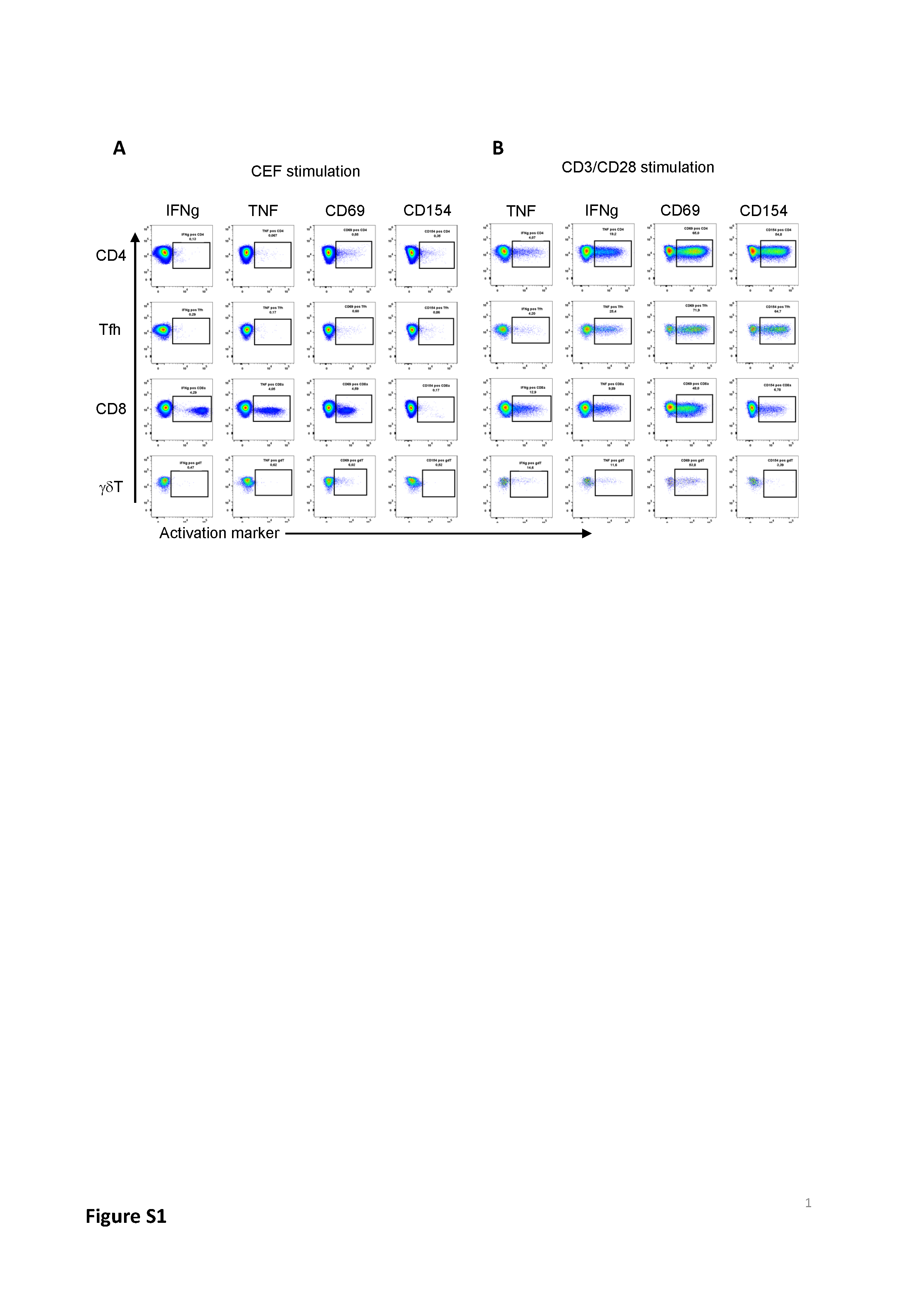

Supplement: Supplementary file 1 [file DataSheet_1.zip › Figure_S1.tiff]

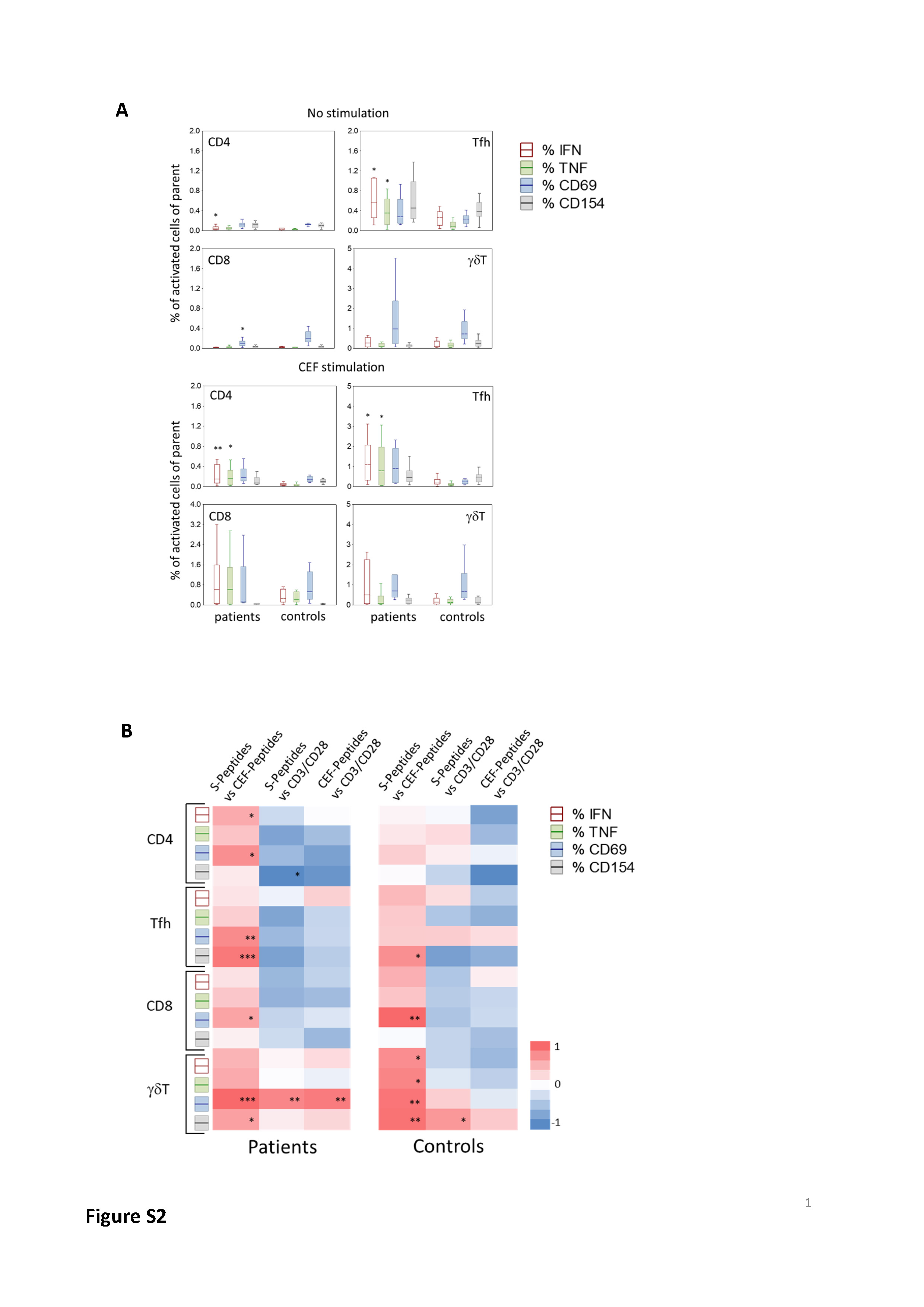

Supplement: Supplementary file 1 [file DataSheet_1.zip › Figure_S2.tiff]

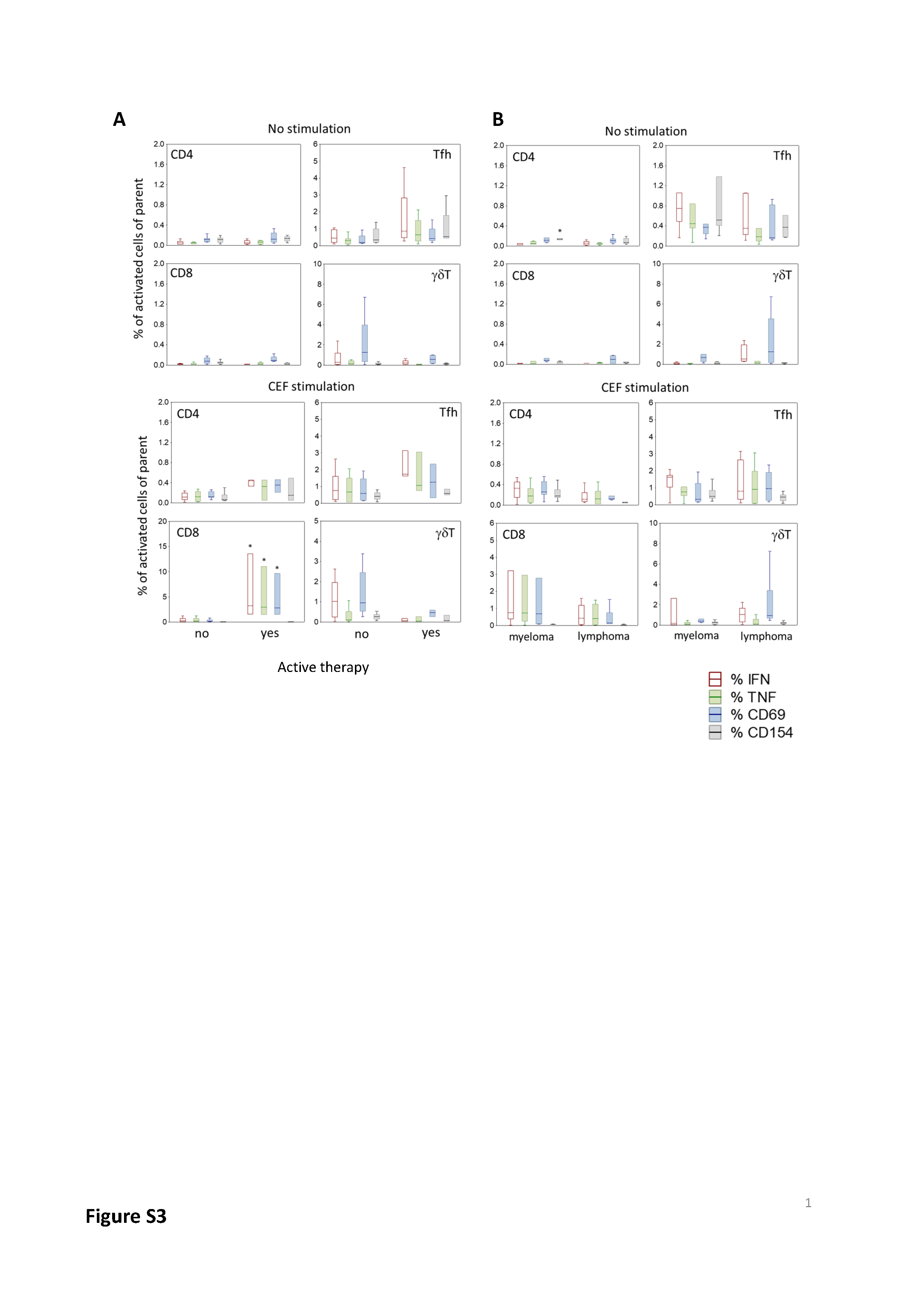

Supplement: Supplementary file 1 [file DataSheet_1.zip › Figure_S3.tiff]
